# Long noncoding RNAs expressed in human hepatic stellate cells form networks with extracellular matrix proteins

**DOI:** 10.1186/s13073-016-0285-0

**Published:** 2016-03-23

**Authors:** Chan Zhou, Samuel R. York, Jennifer Y. Chen, Joshua V. Pondick, Daniel L. Motola, Raymond T. Chung, Alan C. Mullen

**Affiliations:** Gastrointestinal Unit, Department of Medicine, Massachusetts General Hospital, Harvard Medical School, 55 Fruit Street, Boston, MA 02114 USA; Harvard Stem Cell Institute, Cambridge, MA 02138 USA

## Abstract

**Background:**

Hepatic fibrosis is the underlying cause of cirrhosis and liver failure in nearly every form of chronic liver disease, and hepatic stellate cells (HSCs) are the primary cell type responsible for fibrosis. Long noncoding RNAs (lncRNAs) are increasingly recognized as regulators of development and disease; however, little is known about their expression in human HSCs and their function in hepatic fibrosis.

**Methods:**

We performed RNA sequencing and *ab initio* assembly of RNA transcripts to define the lncRNAs expressed in human HSC myofibroblasts. We analyzed chromatin immunoprecipitation data and expression data to identify lncRNAs that were regulated by transforming growth factor beta (TGF-β) signaling, associated with super-enhancers and restricted in expression to HSCs compared with 43 human tissues and cell types. Co-expression network analyses were performed to discover functional modules of lncRNAs, and principle component analysis and K-mean clustering were used to compare lncRNA expression in HSCs with other myofibroblast cell types.

**Results:**

We identified over 3600 lncRNAs that are expressed in human HSC myofibroblasts. Many are regulated by TGF-β, a major fibrotic signal, and form networks with genes encoding key components of the extracellular matrix (ECM), which is the substrate of the fibrotic scar. The lncRNAs directly regulated by TGF-β signaling are also enriched at super-enhancers. More than 400 of the lncRNAs identified in HSCs are uniquely expressed in HSCs compared with 43 other human tissues and cell types and HSC myofibroblasts demonstrate different patterns of lncRNA expression compared with myofibroblasts originating from other tissues. Co-expression analyses identified a subset of lncRNAs that are tightly linked to collagen genes and numerous proteins that regulate the ECM during formation of the fibrotic scar. Finally, we identified lncRNAs that are induced during progression of human liver disease.

**Conclusions:**

lncRNAs are likely key contributors to the formation and progression of fibrosis in human liver disease.

**Electronic supplementary material:**

The online version of this article (doi:10.1186/s13073-016-0285-0) contains supplementary material, which is available to authorized users.

## Background

Liver fibrosis occurs as a result of chronic liver injury and, if left unchecked, often proceeds to cirrhosis and liver failure [1, 2]. Fibrosis develops as the result of accumulation of extracellular matrix (ECM) proteins, including collagen and glycoproteins [3–6], in a process that is driven primarily by transforming growth factor beta (TGF-β) signaling [7, 8]. Hepatic stellate cells (HSCs) are the primary source of the ECM proteins that cause fibrosis [9, 10]. In response to liver injury, quiescent HSCs become activated and produce ECM proteins [9, 11, 12]. When the source of liver injury is removed, activated HSCs revert to an inactive phenotype, resulting in reduced ECM protein expression [13, 14]. In chronic liver disease, the continual activation of HSCs results in differentiation into HSC myofibroblasts and constitutive production of ECM proteins [2]. Collagen is the primary component of the fibrotic scar, and TGF-β is a key signal that promotes collagen expression in HSC myofibroblasts [15–17].

Differentiation of human HSCs into HSC myofibroblasts occurs in vivo in response to chronic liver injury and this process can be modeled ex vivo by growth of HSCs on plastic [9, 11]. Quiescent HSCs are more buoyant than other liver cells due to the presence of fat droplets and can be isolated by density centrifugation [11]. Culture of quiescent HSCs on plastic results in morphological changes and induction of genes, including *ACTA2* (actin, alpha2 smooth muscle), *LOX* (lysyl oxidase), and *LOXL2* (lysyl oxidase like 2), which are characteristic of HSC myofibroblasts [18–21]. Despite an understanding of the protein-coding genes that regulate fibrosis and development of ex vivo tissue culture models to study this process, there are still no effective treatments directed at HSCs to inhibit fibrosis and prevent progression of liver disease.

In recent decades, genome-wide studies have uncovered evidence for extensive transcription outside the regions of DNA that encode proteins [22]. Long noncoding RNA (lncRNA) transcripts are greater than 200 nucleotides (nt) in length and have the same structure as messenger RNAs (mRNAs), including a 5′ cap and a polyadenylated 3′ tail, but do not encode proteins [23]. Over 56,000 lncRNA loci have now been described in human cells [24] and new lncRNAs continue to be identified as new tissues and cell types are analyzed. lncRNAs were originally described as regulators of chromatin [25–27], but as increasing numbers of lncRNAs have been analyzed, it has become clear that they play essential roles in many different cellular processes [28–30]. They are also increasingly recognized as key regulators in mammalian development and disease [30–38], but very little is known about their role in liver fibrosis.

In liver disease, lncRNAs have been studied primarily in relation to cancer. *HULC*, *MALAT-1*, *TUC338*, *TUC339*, *lncRNA-HEIH*, *MVIH*, *HOTAIR*, *lnc-RoR*, and *HOTTIP* have all been associated with higher expression in hepatocellular carcinoma (HCC) compared with normal liver tissue [39–48], while *MEG3* is repressed in HCC [41]. Expression of *MALAT-1*, *HOTAIR*, and *lncRNA-HEIH* was also found to be predictive of HCC recurrence [42, 43, 49] and expression of *HOTTIP* correlates with metastatic HCC burden [46]. *HULC* can be detected in peripheral blood and *TUC339* can be detected in extracellular vesicles, suggesting that each might be able to serve as biomarkers for HCC [39, 50]. Outside of cancer, *lnc-LALR1* is induced in mouse models of liver regeneration, where it promotes hepatocyte proliferation [51]. In addition, *MEG3* is repressed in models of liver injury and in response to TGF-β signaling in the HSC line LX2 [52] and *GAS5* promotes p27 expression to inhibit HSC proliferation and activation [53]. The lncRNAs associated with liver disease were discovered by analyzing the expression of candidate lncRNAs [40, 43, 46] or by screening panels of lncRNAs to identify known lncRNAs that are preferentially expressed in HCC [39, 41, 42, 44, 45, 54]. These studies have not defined the full population of lncRNAs that are expressed in HCC or in liver fibrosis and instead have focused solely on characterizing lncRNAs already described in other cell types.

Many lncRNAs follow cell type-specific patterns of expression [55–57], yet no genome-wide analysis has been performed to identify lncRNAs that are uniquely expressed in HSCs. Thus, we performed RNA-sequencing and ab initio assembly of RNA transcripts to define the lncRNAs expressed in HSC myofibroblasts and those regulated by TGF-β signaling. We analyzed proximity to protein-coding genes, chromatin modifications, response to TGF-signaling, cell type-specific patterns of expression, and clustering by co-expression network analyses in order to identify lncRNAs with the potential to regulate hepatic fibrosis.

## Methods

### Cell culture

Fetal HSCs (Sciencell) were grown in Dulbecco’s modified Eagle medium (DMEM) with 10 % fetal calf serum (FCS) and 1 % penicillin/streptomycin (P/S). Adult human HSCs were isolated from fresh nonparenchymal liver cells obtained from Triangle Research Laboratories. Nonparenchymal cells were centrifuged at 50 × *g* for 5 minutes to remove residual hepatocytes. The cells in the supernatant were pelleted at 860 × *g* for 10 min before re-suspension in Optiprep (Sigma) diluted to 15 % weight per volume (w/v) with Hanks’ balance salt solution without calcium or magnesium. Additional layers of 11.5 % and 8.5 % Optiprep solution were added to the centrifuge tube before centrifugation at 1400 × *g* for 17 min with no brake. HSCs were enriched at the interface between the 11.5 and 8.5 % layers. These cells were removed and expanded in DMEM with 10 % FCS and 1 % P/S. All work with primary human cells was performed with approval of the Massachusetts General Hospital Institutional Review Board (IRB). RNA-sequencing analysis was performed on HSC myofibroblasts after seven to eight passages. Induction of the quiescent-like phenotype was performed by culturing HSCs in growth factor reduced Matrigel (BD). Analysis was performed after 3 days in Matrigel for quantitative RT-PCR. HSCs treated with TGF-β were grown in serum starvation conditions for 48 h in media containing DMEM with 0.2 % bovine serum albumin (BSA) and 1 % P/S. Cells were treated with TGF-β (2.5 ng/ml, R&D systems) for 16 h prior to harvest.

### PCR analysis

RNA was isolated from HSCs using Trizol Reagent (Life Technologies) followed by DNAse I digestion (Life Technologies). RNA was reversed transcribed with Superscript III (Life Technologies). Quantitative RT-PCR analysis was performed with Taqman primer/probe sets (Life Technologies) using the Bio-Rad CFX384 Real Time System. *ACTA2*, *LOX*, *LOXL2*, and *COL1A1* expression was normalized to *GAPDH*. The following Taqman primer/probe sets were used: Hs_00426835, *ACTA2*; Hs_00942480, *LOX*; Hs_00158757, *LOXL2*; Hs_00164004, *COL1A1*; Hs_02758991, *GAPDH*.

### Microscopy

Matrigel (200 μl, BD) was distributed across the surface of each well of a 24-well plate and allowed to gel before HSC myofibroblasts were added to the well. Activated HSCs were plated on plastic. After 3 days, the medium was aspirated, and Bodipy 493/503 (Life Technologies) and Hoescht were diluted in media and added to the wells. Bodipy was added at a concentration of 67 pg/μl and Hoescht was added at a concentration of 5 pg/ul. After 45 minutes, the cells were washed twice with Dulbecco's phosphate-buffered saline (DPBS) and imaged with a Nikon A1plus confocal microscope 10× lens. The cells were observed using a pinhole setting of 255.4 μm. Laser intensity, background level, contrast, and electronic zoom size were collected at the same level for each experiment. Image processing was performed using Adobe Photoshop software.

### Preparation of RNA-seq and ChIP-seq libraries

Total RNA was isolated using Trizol reagent followed by clean up using either the MirVana® Isolation Kit (Life Technologies) following instructions for total RNA isolation or re-precipitation after phenol:chloroform and choloroform extractions. RNA quality was assessed via Agilent 2100 Bioanalyzer and samples with RNA integrity numbers (RIN) greater than or equal to 9 were used for library preparation. Isolated RNA was prepared for sequencing according to TruSeq Stranded mRNA Library Prep Kit (Illumina). Chromatin immunoprecipitation (ChIP) was performed using antibodies to detect enrichment of H3K4me3 (07-472, Millipore) and H3K27ac (Ab4729, Abcam) as previously described [58] with the following modifications: 1 × 10^7^ cells were sheared for 5 min in 1 ml of cell lysis buffer (Covaris) using a Covaris S220 set to a peak wattage of 140, duty factor 5 % and 200 cycles per burst; immunoprecipitations were performed using 1 μg of antibody with 10 μl of magnetic beads. ChIP-seq libraries were prepared using the TruSeq ChIP Sample Prep Kit (Illumina). They were sequenced using the Illumina HiSeq 2000 to obtain 100 × 100-nt paired-end reads for fetal HSCs and used for ab initio assembly (see below). Illumina HiSeq 4000 was used to obtain 50-nt single-end reads for primary adult HSC myofibroblasts. We used 50-nt single-end reads for ChIP-seq analysis.

### Ab initio assembly of transcripts from RNA-seq data

We mapped each replicate of directional paired-end RNA-seq data to the human reference genome (hg19/GRCh37) using TopHat v2.0.10 [59, 60] before assembling transcripts using both Cufflinks [61] and Scripture [62]. The TopHat settings were as follows:

*tophat -p 8 --library-type fr-firststrand --mate-inner-dist 50 --mate-std-dev 50 --microexon-search --GTF genes.gtf -o < output-folder > <index of reference genome > Reads_end1.fastq Reads_end2.fastq*

The reference genes in GTF file format (genes.gtf) were downloaded from the University of California, Santa Cruz (UCSC) genome browser [63]. We then assembled transcripts through the following settings of Cufflinks using TopHat output bam file as input:

*cufflinks -p 8 --max-bundle-frags 100000000 --library-type fr-firststrand --frag-bias-correct --multi-read-correct -o < output_folder > <tophat_output_bam_file>*

Max-bundle-frags was set to 100,000,000 such that highly expressed genes would be included in the output.

For analysis in Scripture, we used the following TopHat settings:

*tophat -p 4 --microexon-search --GTF genes.gtf -o < output_folder > <index_of_reference_genome > <Reads_one_end.fastq>*

We used Scripture (beta2 version) to assemble transcripts by following the protocol for transcript assembly (http://www.broadinstitute.org/software/scripture/). All transcripts assembled in Cufflinks and/or Scripture were then merged into one list through Cuffmerge [61].

### Identification of long noncoding RNAs

The assembled transcripts were then filtered through the following steps to identify lncRNAs:

1. Removed transcripts that overlapped with annotated protein-coding genes, pseudogenes, rRNAs, tRNAs, small nucleolar RNAs (snoRNAs) and microRNAs on the same strand.
2. Removed transcripts with protein coding potential. The coding potential of each remaining transcript was estimated by HMMER protein domain search [64, 65] and CPAT [66] using an alignment-free logistic regression model. The Pfam protein families database (v27.0) was downloaded from EMBL-EBI [67]. Both Pfam-A, containing high-quality and manual curated families, and Pfam-B, containing automatically generated comprehensive protein families, were used in the HMMER domain search. We removed the transcripts matching a protein domain with *p* value <1e-4. CPAT uses a logistic regression model built with four sequence features: open reading frame size, open reading frame coverage, Fickett TESTCODE statistic, and hexamer usage bias to estimate the coding ability of transcripts. We used 0.364 as the threshold for discriminating noncoding and coding transcripts. This threshold (0.364) was chosen because it gives the highest sensitivity and specificity (0.966 for each) for human data [66] according to the nonparametric two-graph receiver operating characteristic (ROC) curves.
3. Removed remaining transcripts that overlapped on the same strand with any transcripts removed in steps 1 or 2.
4. Removed remaining transcripts that lacked H3K4me3 occupancy within 1 kb of their 5′ end.
5. Removed remaining transcripts that were shorter than 200 nt or had low read coverage as defined as less than 0.01 reads per kilobase per million unique mapped reads or less than ten reads per transcript.

### ChIP-Seq analysis for HSCs

ChIP-seq datasets were aligned to the human reference genome (hg19/G37) using Bowtie2 [68]. We performed alignment in end-to-end alignment mode with the settings “*bowtie2 -k2 -N1 -L32 --end-to-end*”. We next used the MACS2 [69] *callpeak* function to compare the mapped bam files of each ChIP to its matched whole cell extract background control. H3K4me3 and H3K27ac peaks were predicted using the following setting: *-q 0.01 --nomodel --shiftsize* = *150*. This setting was used because histone marks have an underlying characteristic fixed resolution for nucleosome size and our ChIP-seq only sequenced 50 nt at the 3′ end. SMAD3 ChIP-seq data from LX2 cells were downloaded from the Gene Expression Omnibus (GEO; accession GSM934613 and GSM934616) and SMAD3 peaks were called by using the default settings in MACS2. We defined genes as bound by SMAD3 if the site of occupancy was within 10 kb upstream of the transcription start site (TSS) or within the gene body.

### Identification of super-enhancers

H3K27ac peaks that were called using MACS2 were analyzed using ROSE [70, 71] to classify enhancers into typical enhancers and super-enhancers based on H3K27ac signals. The human reference genome (build hg19) and H3K27ac peaks were used as input files. lncRNAs were considered to be associated with super-enhancers or typical enhancers if the enhancer was located within 10 kb of the lncRNA TSS.

### Classification of lncRNAs

We classified lncRNA loci into four categories according to their genomic locations. An lncRNA was classified as divergent if the TSS of the lncRNA locus was within 2 kb of the TSS of a protein-coding gene on the opposite strand. Any remaining lncRNAs that were antisense to a protein-coding gene and overlapped the protein-coding gene by one or more base pairs were classified as natural antisense. Remaining lncRNAs located within a 1000-bp window of a region of H3K27ac occupancy were classified as enhancer-associated. Any remaining lncRNAs that had a TSS greater than 2 kb from the TSS of the nearest protein-coding gene were classified as intergenic.

### Calculation of expression levels for protein-coding genes and lncRNAs

The expression levels of all protein-coding genes and lncRNA loci, represented in fragments per kilobase of transcript per million fragments mapped (FPKM), were calculated by Cuffdiff (v2.2.1) with the following parameters: “*--max-bundle-frags 100000000 --library-type fr-firststrand -b < hg19 reference genome > --multi-read-correct --no-effective-length-correction --min-isoform-fraction 0 --min-alignment-count*”. The max-bundle-frags setting was increased from the default parameters so that highly expressed genes would not be excluded.

### Differential expression analyses

To increase the sensitivity in detecting changes in expression of protein-coding and lncRNA genes, two approaches were used to quantify changes in expression: (a) TopHat [59, 72] and Cuffdiff (v2.2.1) [61] and (b) HTSeq [73] and DESeq2 [74]. We took the union of differential expressed lncRNAs found either by Cuffdiff or DESeq2 (adjusted *p* < 0.05). We used the FPKM values to quantify changes in expression for all figures unless otherwise stated.

### Comparison of lncRNAs and protein-coding gene structure

To compare the number of exons, transcript lengths, and gene lengths between lncRNAs in HSCs and protein-coding genes, the longest isoform for each locus was selected to represent an lncRNA or protein-coding gene. All protein-coding genes expressed in HSCs were used for these comparisons.

### Raw RNA-seq data of other human tissues and cell types

We obtained the raw RNA-seq data for 37 human tissues (see Additional file 1: Table S1 for tissue name and GTEx ID) and dermal fibroblasts from dbGaP [75]. RNA-seq data from the six tier 1 and tier 2 Encyclopedia of DNA Elements (ENCODE) cell lines were downloaded from GEO. GM12878, K562, HeLa-S3, HepG2, and human umbilical vein endothelial cell (HUVEC) data were obtained from GSE26284 and H1 (WA01) human embryonic stem cell data from GSE41009. RNA-seq data of pancreatic stellate cells and immortalized human induced fibroblast (hiF-T) cells were downloaded from GSE43770 and GSE62777, respectively.

### Analysis of lncRNA expression across tissues and cell types

Three samples were selected from each of 37 distinct human tissues from the Genotype-Tissue Expression (GTEx) project [75] and downloaded from dbGaP. The HSC myofibroblasts analyzed in this study were male, so the two male and one female samples with the highest number of reads were selected for each tissue (Additional file 1: Table S1). Where tissues were male- or female-specific, all three samples were selected from the same sex. The FPKM values for protein-coding and lncRNA genes were used to quantify changes in expression. The Wilcoxon–Mann–Whitney test was used to identify the lncRNAs overexpressed in HSC myofibroblasts and HSC myofibroblasts treated with TGF-β compared with 37 human tissues and six ENCODE cell lines. The dendrogram for clustering the human samples was calculated by the default setting of the *R* function *heatplot* (in the gplots and made4 libraries of *R*).

RNA-seq data from 35 liver samples from GTEx [75] were downloaded from dbGaP. The pathology reads were obtained from GTEx. Of the 35 liver samples, eight showed normal histology, two showed bridging fibrosis, and two showed cirrhosis (Additional file 2: Table S2). These 12 samples were selected for further analysis. The Z-score was calculated by subtracting the mean expression of an lncRNA for each row from the individual expression level of the lncRNA in a sample and dividing by the standard deviation.

### Generation of BigWig files

Fetal HSC RNA-seq data and the RNA-seq data of six ENCODE cell types are directional, paired-end data and both paired-end reads were mapped independently to the reference genome (hg19) in order to retain strand-specific information for HSC and ENCODE RNA-seq data. In this way the second-strand sequence was mapped to the genome and the reverse complement of the first-strand sequence was mapped to the genome. We then converted the mapped reads with strand information into BigWig files.

The RNA-seq data from GTEx is undirectional paired-end data, so we mapped both end reads into the human reference genome without strand information. BigWig files for ENCODE and GTEx were generated using TopHat to align to the genome with the setting “–N 0”, which was necessary to exclude a peak in this region that mapped to multiple genomic locations.

We used the “*bdgcmp*” function of MACS2 to subtract the whole-cell extract background sequencing reads from the ChIP-seq reads using the log-likelihood ratio (logLR) method, which calculates the log10 likelihood ratio between the ChIP and whole-cell extract. All ChIP and whole-cell extract background sequencing reads were normalized by their sequencing depths. We then converted the mapped reads into BigWig files.

### Co-expression analysis and network construction

We constructed the co-expression networks for lncRNAs and all protein-coding genes from RefSeq (version of 10 Feb 2014) using the *mcxarray* program in the Markov Clustering (MCL)-edge network analysis tool [76] (http://micans.org/mcl/) with Spearman correlation. Gene expression was calculated using Cuffdiff (see the previous section in the “*Methods*” for details). In this study, we choose 0.7 as the Spearman correlation cutoff in order to balance the number of singletons and the median node degree as recommended by the MCL protocol. To make the co-expression analysis consistent through the entire project, we used the same correlation and threshold to examine if the divergently transcribed coding genes and lncRNAs are co-expressed.

We adopted the MCL algorithm to identify clusters from the constructed large networks. The MCL algorithm is coded in the *mcl* program [76], which is a fast and scalable unsupervised cluster algorithm for networks based on simulation of stochastic flow in networks. The default granularity “–I 1.4” was set for MCL clustering. Cytoscape v3.1.1 [77] was used to visualize the connected networks and clusters.

For the nucleotide-binding module, all the protein-coding genes annotated in the Gene Ontology (GO) nucleotide binding category in cluster I and their directly co-expressed lncRNAs (linking by one-edge) in cluster I were selected to be displayed. For the extracellular matrix module, all the protein-coding genes annotated in the GO extracellular matrix category in cluster II and their directly co-expressed lncRNAs (linking by one-edge) in cluster II were selected to be displayed.

### GO enrichment analyses

GO enrichment analysis was performed using the protein-coding genes identified in each co-expression cluster (http://david.abcc.ncifcrf.gov/) [78, 79]. All protein-coding genes used in the network construction were used as the background in the GO enrichment analyses.

### Statistical analysis

The *p* values in this study were calculated by Wilcoxon–Mann–Whitney test, unless otherwise mentioned.

### Principal component analysis

We used pricipal component analysis (PCA) to examine the similarity in expression patterns of lncRNAs among the indicated cell types and to examine the similarity in expression patterns of lncRNAs plus protein coding genes between the same cell types. Each replicate for each sample is represented with a vector of the expression levels of the indicated genes. All principal components (PCs) were identified for each replicate through PCA on its expression vector. We then used the k-mean method to cluster the replicates of all samples according to the first three major components. Graphs display the first and second major components (PC1 and PC2) or the first and third major components (PC1 and PC3) in two dimensions.

### Identification of enhancer-RNAs

To identify the enhancer-RNAs (eRNAs), we downloaded the H3K4me3 and H3K4me1 ChIP-seq data for hiF-T cells (GSE62777). We excluded lncRNAs expressed in HSC myofibroblasts that were classified as divergent or natural antisense from eRNA analysis because their histone marks cannot be distinguished from marks of neighboring genes [80]. For the remaining lncRNAs, we selected those that are also expressed in hiF-T cells according to their calls by Cuffdiff. Next, we mapped the raw H3K4me3 and H3K4me1 reads to the TSS regions of each lncRNA (defined as within ±500 bp of the TSS) and then calculated the H3K4me1/H3K4me3 ratio for each lncRNA. A ratio of >1.2 was used to define eRNAs, as previously described [80].

### Data access

RNA-seq and ChIP-seq data produced for this study are available in the GEO (accession GSE68108).

## Results

### De novo identification of lncRNAs in human HSCs

**Fig. 1:**
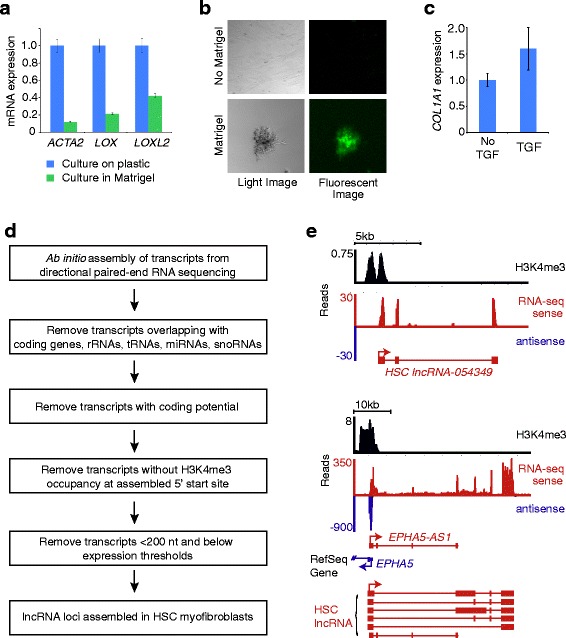
Identification of lncRNAs in human HSC myofibroblasts. **a** *ACTA2*, *LOX*, and *LOXL2* mRNA levels were quantified in HSC myofibroblasts cultured on plastic (*blue*) and compared with mRNA levels in HSC myofibroblasts that were cultured in Matrigel for 3 days to induce reversion to an inactive phenotype (*green*). Samples were normalized using *GAPDH*, and expression of each gene in HSC myofibroblasts was set to 1. *Error bars* represent standard deviation. This experiment is representative of three biological replicates. **b** Culture in Matrigel leads to accumulation of fat droplets characteristic of HSCs that have reverted to an inactive phenotype. Microscopy images are shown for HSC myofibroblasts cultured without (*No Matrigel*) and with Matrigel for 3 days. Light microscopy images (*left*) show that the cells grown in Matrigel become rounded and clump together. Bodipy staining for neutral fat (*green fluorescence*, *right*) shows accumulation of lipid droplets in HSCs cultured in Matrigel. Images are shown at 10× magnification. **c** *COL1A1* mRNA levels were quantified in HSC myofibroblasts cultured in 0.2 % bovine serum albumin and after treatment with TGF-β for 16 h. **d** Pipeline for identification of lncRNAs (see the “*Methods*” for details). *miRNA* microRNA. **e** Examples of lncRNAs identified in HSC myofibroblasts. *lncRNA-054349* is a novel lncRNA discovered in HSCs (*top*) and *EPHA5-AS1* is an lncRNA expressed in HSCs that is expressed antisense to *EPHA5* (*blue*). For each lncRNA track, H3K4me3 enrichment (log likelihood ratio (LogLR) value normalized by subtracting the background) marks the site of transcription initiation (*black*, *top*). RNA-seq reads supporting lncRNA transcripts are shown in *red* (*RNA-seq sense*). Antisense transcripts are shown in *blue*. The genomic structure for each gene is shown below the RNA-seq tracks. lncRNAs are shown in *red* and protein-coding genes are shown in *blue. Boxes* indicate exons, *lines* indicate introns, and *arrows* indicate the start and direction of transcription. For *EPHA5-AS1*, the annotated lncRNA and protein-coding gene are shown below the RNA-seq tracks. The multiple isoforms of *EPHA5-AS1* that are identified in HSC myofibroblasts (labeled as *HSC lncRNA*) are shown at the *bottom*. Novel lncRNAs identified in this study are named with the prefix “*lncRNA*” followed by the locus number assigned during assembly

We first established that primary human fetal HSC myofibroblasts share characteristics described for HSCs. These cells were chosen for initial analysis as they could be expanded more easily for genome-wide sequencing. Culture in Matrigel repressed *ACTA2*, *LOX*, and *LOXL2* expression and induced accumulation of lipid droplets characteristic of the reversion to an inactive HSC phenotype in Matrigel (Fig. 1a, b) [81, 82]. These HSCs also showed induced collagen expression in response to TGF-β signaling (Fig. 1c) [15]. We then performed massively parallel sequencing of polyadenylated RNAs (RNA-seq) to identify lncRNAs that are expressed in human HSC myofibroblasts. Directional libraries were prepared for paired-end sequencing so that overlapping sense and antisense transcripts could be distinguished from one another during analysis. We then established a computational pipeline for ab initio construction of lncRNA transcripts (Fig. 1d) [58, 62, 83]. RNA-seq reads were first aligned to the genome with TopHat [59, 60]. Cufflinks [61] and Scripture [62] were each used to assemble the aligned reads into transcripts, which were then merged by Cuffmerge [60, 61]. All assembled transcripts that overlapped on the same strand with protein-coding genes in RefSeq [84] or the UCSC database [85] were removed, as well as those overlapping with rRNAs, tRNAs, microRNAs and snoRNAs annotated in RefSeq. The remaining transcripts were analyzed by a HMMER profile search of protein domains [64, 65] and CPAT software with a logistic regression model [66] to assess their protein-coding potential (Additional file 3: Figure S1). Transcripts that had low protein-coding potential as predicted by both HMMER and CPAT were considered to be noncoding RNAs. We next performed ChIP-seq using an antibody against the chromatin mark H3K4me3 to identify sites of transcription initiation [86]. All RNA transcripts whose 5′ ends were greater than 1 kb from an H3K4me3 site were removed because the absence of a proximal transcription initiation mark suggested that the assembled RNA transcripts might be incomplete. All transcripts less than 200 nt in length and below expression thresholds were removed (see “*Methods*” for additional details). We identified and assembled 7189 lncRNA transcripts into 2808 lncRNA loci (Additional file 4: Table S3).

Two lncRNAs identified with this pipeline are shown in Fig. 1e. *lncRNA-054349* (top) is an example of a three-exon lncRNA that was not previously annotated and *EPHA5-AS1* is an example of a lncRNA for which a single isoform was previously annotated. For both lncRNAs, H3K4me3 occupancy is shown in black (top) and the RNA-seq reads supporting the lncRNAs are shown in red (sense). The genomic structure of each lncRNA is indicated below the tracks in red, with arrows indicating the direction of transcription. *EPHA5-AS1* is divergently transcribed from *EPHA5* and the RNA-seq reads supporting *EPHA5* transcripts are indicated in blue (antisense).

### Genomic characterization of lncRNAs

**Fig. 2:**
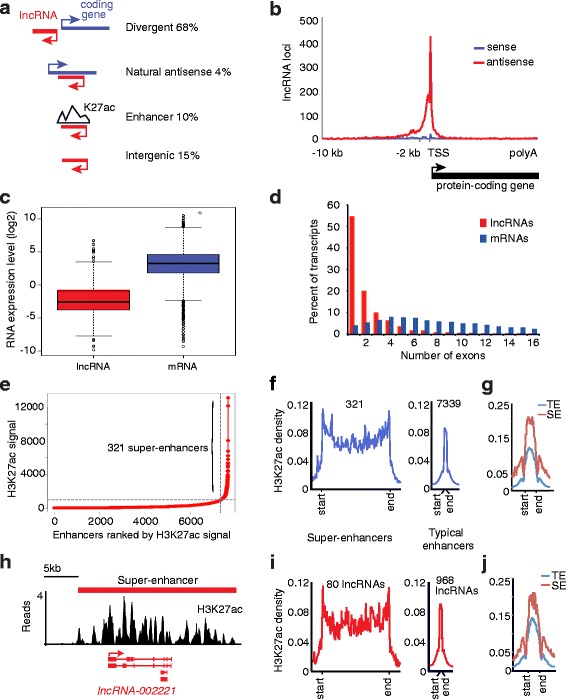
Genomic and expression features of HSC lncRNAs. **a** Classification of lncRNA loci identified in HSC myofibroblasts. Divergent lncRNAs are defined as having a transcription start site (*TSS*) within 2 kb of the TSS of a protein-coding gene and being transcribed from the strand antisense to the protein-coding gene. Natural antisense lncRNAs overlap the coding gene by at least one base pair. Enhancer-associated lncRNAs are located within 1 kb of an enhancer as defined by H3K27ac. Intergenic lncRNAs have a TSS greater than 2 kb from the TSS of the nearest protein-coding gene and are not contained in any of the other categories. lncRNAs are indicated in *red* and protein-coding genes are indicated in *blue. Arrows* show the start and direction of transcription. H3K72ac (*K27ac*) peaks mark enhancers. The abundance of each class of lncRNA is displayed on the *right*. **b** Distribution of the TSS of lncRNAs relative to the TSS of their nearest protein-coding gene. All protein-coding genes were normalized to equal length on the *x-axis* (*black rectangle*), and 10 kb of genomic sequence upstream of the TSS of each protein-coding gene is shown. The location of the TSS of each lncRNA was plotted relative to the TSS of the nearest protein-coding gene. lncRNAs that are antisense to protein-coding genes are indicated in *red* and lncRNAs that are sense to protein-coding genes are indicated in *blue*. The *red peak* near the protein-coding TSS indicates that the majority of lncRNAs are located within 2 kb of protein-coding genes and transcribed antisense to protein-coding genes. **c** lncRNAs (*red*) are expressed about tenfold lower than their divergent protein-coding genes (*blue*). The expression levels (log2 transformed reads per fragment per million mapped reads (FPKM)) are indicated on the *y-axis*. The *horizontal black line* indicates the mean and *open circles* indicate outliers. **d** lncRNAs contain fewer exons than protein-coding genes. The distribution of exons in lncRNA (*red*) and mRNA (*blue*) transcripts. **e** Identification of super-enhancers in HSC myofibroblasts. Enhancers were defined by H3K27ac occupancy and ranked from *left* to *right* (*x-axis*) by the total reads of H3K27ac mapped to each enhancer (*H3K27ac signal*, *y-axis*). We identified 321 super-enhancers from a total of 7660 enhancers [70]. **f** Super-enhancers show broad domains of occupancy compared with typical enhancers. Metagenes represent the mean H3K27ac density (in reads per million unique mapped reads per base pair) across super-enhancers (*left*) and typical enhancers (*right*). The metagenes are centered on the enhancer region for each plot and display 3 kb of sequence flanking each enhancer. The median size of a super-enhancer is 18,807 bp and the median size of a typical enhancer is 678 bp. The plots are scaled (*x-axis*) to reflect the median size of the two classes of enhancers. The increase of signal at the boundary is characteristic of super-enhancers [71] because the boundary represents the H3K27ac peaks at the edges of each super-enhancer. Thus, the peaks at the boundary tend to be aligned with each other while peaks away from the boundaries are distributed more equally. **g** H3K27ac peaks are enriched at super-enhancers. Metagenes represent the mean H3K27ac density across the major peak of super-enhancers (*SE*) and typical enhancers (*TE*). **h** Example of an lncRNA associated with a super-enhancer. H3K27ac occupancy (normalized logLR value, *y-axis*) is shown surrounding *lncRNA-002221* (*red*). The domain of the super-enhancer is indicated by a *red rectangle*. **i** Super-enhancers were found to be associated with 80 lncRNAs. The median size of super-enhancers associated with lncRNAs was 18,699 bp and the median size of typical enhancers associated with lncRNAs was 1032 bp. We located 968 lncRNAs within 10 kb of a typical enhancer. The enhancers were plotted as described in **f**. **j** H3K27ac peaks are enriched at super-enhancers associated with lncRNAs. Metagenes represent H3K27ac density across the major peak of super-enhancers and typical enhancers associated with lncRNAs

We next assessed the distribution of lncRNAs across the genome. Over 65 % of HSC lncRNAs were divergently transcribed from the promoter regions of protein-coding genes. Four percent of the total identified lncRNAs were antisense to coding genes (natural antisense), 10 % were located at enhancers as defined by the presence of H3K27ac [87], and 15 % originated from intergenic regions away from coding genes (Fig. 2a). Plotting the location of the transcriptional start site (TSS) of each lncRNA relative to the TSS for the closest protein-coding gene showed that the vast majority of lncRNAs are transcribed antisense to protein-coding genes and originate near the promoter of these genes (Fig. 2b), with the TSS of the lncRNAs located a mean distance of 135 nt upstream of the TSS of the paired protein-coding genes. We also found that lncRNAs in HSCs tend to be expressed at lower levels than protein-coding genes. Analysis of the expression level of lncRNAs and their divergently transcribed mRNAs showed that lncRNAs are expressed at approximately tenfold lower levels than protein-coding genes (Fig. 2c; *p* < 2.2e-16). These findings are consistent with the description of lncRNAs in other cell types [55, 56, 58, 88].

Most of the identified lncRNA transcripts (>55 %) are single exon transcripts whereas only 4 % of protein-coding genes expressed in HSC myofibroblasts are encoded by single exons (Fig. 2d). HSC lncRNAs also tend to have shorter genes and shorter transcripts than protein-coding genes (Additional file 3: Figure S2) [88].

Super-enhancers are large domains of active enhancers associated with genes that control cell identity [70, 71]. To identify lncRNAs that may regulate key facets of HSC identity and function, we asked if lncRNAs are associated with super-enhancers in HSCs. We performed ChIP-seq to identify regions of chromatin containing the histone modification H3K27ac, which is enriched at active enhancers [87]. We identified 7339 typical enhancers and 321 super-enhancers (Additional file 5: Table S4) according to their H3K27ac signal strengths (Fig. 2e). These super-enhancers are an order of magnitude larger than typical enhancers in genomic coverage (Fig. 2f) and have larger peaks of H3K27ac enrichment than typical enhancers (Fig. 2g). Super-enhancers are also associated with lncRNAs identified in HSCs; for example, *lncRNA-002221* is located within a super-enhancer that spans greater than 20 kb (Fig. 2h). Eighty lncRNAs discovered in HSC myofibroblasts were found to be associated with super-enhancers. H3K27ac signal at these super-enhancers associated with lncRNAs also shows increased genomic coverage (Fig. 2i) and larger peaks than typical enhancers (Fig. 2j).

### TGF-β signaling directly regulates lncRNA expression in HSC myofibroblasts

**Fig. 3:**
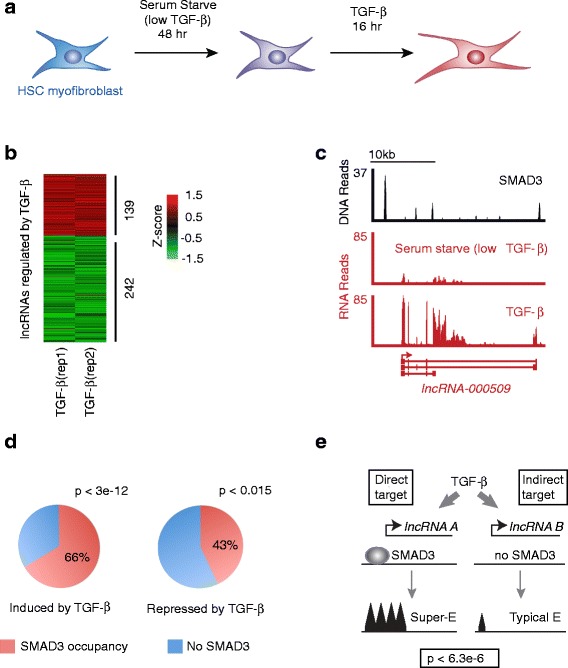
Regulation of HSC lncRNAs. **a** Conditions for HSC lncRNA analysis. HSC myofibroblasts were cultured for 48 h in 0.2 % bovine serum albumin to remove exogenous TGF-β from the media (low TGF-β) and then treated with TGF-β for 16 h to define lncRNAs regulated by TGF-β signaling. **b** lncRNA expression in response to TGF-β signaling. The heatmap shows normalized expression of lncRNAs that were induced (*red*) or repressed (*green*) in response to TGF-β signaling. The Z-score is shown on the *right* and was calculated from expression of lncRNAs in two replicates of low TGF-β conditions (not shown) and two replicates of TGF-β treatment (shown). Each *row* represents one lncRNA locus and lncRNA loci are ranked by *p* value such that the smallest *p* value of induced lncRNAs is at the *top* and the smallest *p* value of repressed lncRNAs is at the *bottom*. **c** The gene encoding *lncRNA-000509* is occupied by SMAD3 and induced in response to TGF-β signaling. SMAD3 occupancy [90] is shown at the *top*. RNA-seq reads (*red*) are displayed for HSCs in low TGF-β and after 16 h of TGF-β signaling. The structure and direction of *lncRNA-000509* is shown below. **d** lncRNAs that are regulated by TGF-β signaling tend to be occupied by SMAD3. The fraction of lncRNA genes that are induced (*left*) and repressed (*right*) by TGF-β and are occupied by SMAD3. Genes that are regulated in response to TGF-β signaling and occupied by SMAD3 are considered direct targets of TGF-β signaling. **e** lncRNAs directly targeted by TGF-β signaling tend to be associated with super-enhancers. lncRNA genes that change in expression in response to TGF-β signaling and are occupied by SMAD3 (*Direct target*) are associated with super-enhancers (*Super-E*), while lncRNAs that change in expression in response to TGF-β signaling and are not directly occupied by SMAD3 (*Indirect target*) are associated with typical enhancers (*Typical E*)

TGF-β is a key regulator of fibrosis in liver disease [15–17] and we next asked if TGF-β signaling regulates expression of lncRNAs in HSC myofibroblasts. HSC myofibroblasts were serum starved for 48 h to remove exogenous TGF-β from the media, followed by treatment with TGF-β for 16 h (Fig. 3a). RNA was harvested from HSC myofibroblasts that were treated with TGF-β after serum starvation and from HSC myofibroblasts that remained in serum starvation conditions. We performed RNA-seq analysis to confirm that TGF-β treated HSCs respond to TGF-β signaling (Additional file 3: Figure S3a) and then used our computational pipeline (Fig. 1d) to assemble the lncRNAs expressed during serum starvation (low TGF-β) and after TGF-β treatment. Wer assembled 2078 lncRNA loci in serum-starved HSC myofibroblasts and 1759 lncRNA loci after TGF-β treatment (Additional file 3: Figure S3b).

The lncRNA transcripts defined in HSC myofibroblasts, serum-starved HSC myofibroblasts, and TGF-β-treated HSC myofibroblasts were combined using Cuffcompare [60, 61] to define a total of 16,299 lncRNA transcripts expressed in at least one condition. These transcripts were classified into 3692 lncRNA loci and represent all the lncRNA loci detected in human HSCs (Additional file 6: Table S5 and Additional file 7: Table S6). Many of these lncRNAs are novel, including 40 % that do not overlap with a single nucleotide of human lncRNAs annotated in existing databases (Additional file 3: Figure S3c).

We identified 139 lncRNA loci that were induced and 242 lncRNA loci that were repressed with activation of TGF-β signaling (Fig. 3b; *p* < 0.05). The transcription factors SMAD2 and SMAD3 are activated by TGF-β signaling to mediate the transcriptional effects of the canonical TGF-β signaling pathway [89] and SMAD3 appears to play the dominant role in HSC myofibroblast activation [12]. To determine the direct targets of TGF-β signaling, we identified the lncRNAs that were regulated by TGF-β and occupied by SMAD3. For example, *lncRNA-000509* is occupied by SMAD3 and shows a tenfold induction between serum starvation (low TGF-β) and induction of TGF-β signaling (Fig. 3c). Sixty-six percent of lncRNAs induced by TGF-β (*p* < 3e-12) and 43 % of lncRNAs repressed by TGF-β (*p* < 0.015) were found to be occupied by SMAD3 in the human HSC line LX2, following TGF-β treatment [90] (Fig. 3d). This analysis identified 91 lncRNAs that are directly induced by TGF-β signaling and 104 lncRNAs that are directly repressed by TGF-β signaling, with a mean induction and repression of twofold (Additional file 8: Table S7).

TGF-β signaling targets cell type-specific enhancers bound by master transcription factors [91] and super-enhancers regulate genes that define cell identity [70, 71]. We also found that lncRNAs directly targeted by TGF-β signaling (regulated by TGF-β and occupied by SMAD3) are more likely to be associated with super-enhancers in HSCs than lncRNAs indirectly targeted by TGF-β signaling (Fig. 3e; *p* < 6.3e-6), suggesting that these lncRNAs may also play a key role in cell identity. Coding genes directly targeted by TGF-β signaling are also more likely to be associated with super-enhancers than indirectly targeted genes (*p* < 5.9e-14). However, lncRNAs that are directly targeted by TGF-β signaling are more likely to be regulated by super-enhancers than coding genes directly targeted by TGF-β (*p* < 1.6e-13).

### lncRNAs enriched in HSC myofibroblasts

**Fig. 4:**
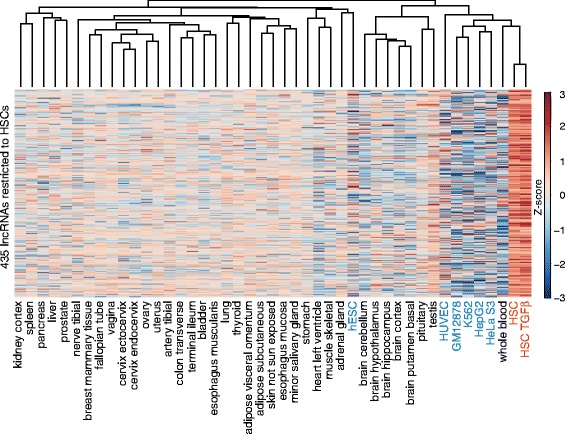
Expression of HSC-specific lncRNAs. The normalized expression levels are shown for 435 lncRNAs (*y-axis*) that are enriched in HSCs compared with other cell types and tissues. RNA-seq data from HSC myofibroblasts and HSC myofibroblasts treated with TGF-β (*red*) were compared with 37 normal tissues (*black text*) [75] and six ENCODE cell lines (*blue text*) [92]. The *dendrogram* at the *top* indicates hierarchical clustering. Each *row* represents one lncRNA locus. *Red shading* indicates induction and *blue shading* indicates repression relative to the levels of each lncRNA across all tissues. The Z-score is shown on the *right*. lncRNAs were considered HSC-specific if they were enriched in HSCs compared with other tissues with a *p* value of <0.05 (Wilcox–Mann–Whitney test)

**Fig. 5:**
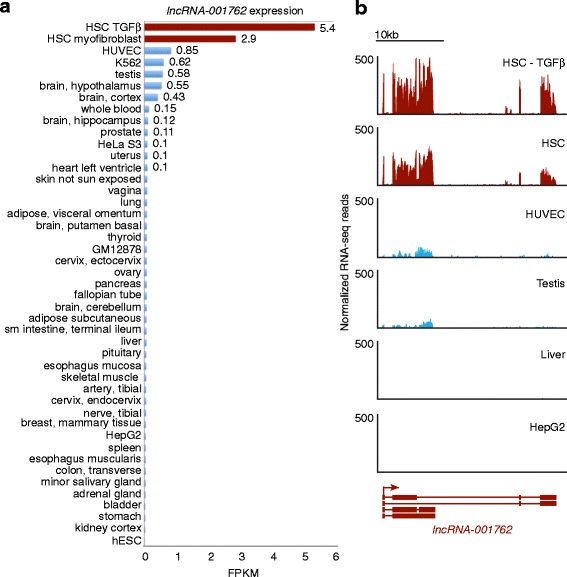
*lncRNA-001762* is restricted in expression to HSCs. **a** Expression of *lncRNA-001762* was quantified by FPKM in each cell type (*y-axis*). The highest level of expression was found in HSCs treated with TGF-β. **b** RNA-seq reads (*y-axis*) were normalized and displayed across the gene encoding *lncRNA-001762.* Expression was compared with the cell line (*HUVEC*) and tissue type (*Testis*) that show the highest levels of *lncRNA-001762* expression outside HSCs. Expression was also visualized in primary liver tissue and the hepatocellular carcinoma line HepG2. The structure and direction of transcription for *lncRNA-001762* is shown below

lncRNAs appear to be under weaker evolutionary constraints than protein-coding genes, resulting in more cell type-specific patterns of expression compared with coding genes [55–57]. While approximately 40 % of the 3692 lncRNA loci identified in HSCs were not described in other lncRNA databases (Additional file 3: Figure S3c), it is unclear if this result means that these lncRNAs are unique to HSCs or that many of these lncRNAs are expressed in other cell types but have not been described. To define the lncRNAs specific to HSCs, we examined the expression patterns of lncRNAs identified in HSC myofibroblasts and HSC myofibroblasts treated with TGF-β compared with 37 other human tissues [75] and the six tier 1 and tier 2 cell lines from the ENCODE project [92]. Analyses revealed that more than 400 lncRNAs are significantly enriched in HSCs compared with the 43 other tissues and cell types analyzed (Fig. 4; *p* < 0.05; Additional file 9: Table S8). This enrichment is observed relative to primary tissue, including liver, and six cell lines, including the hepatocellular carcinoma cell line HepG2. For example, *lncRNA-001762* is expressed at greater than sixfold higher levels in HSC myofibroblasts treated with TGF-β compared with the highest level of expression in 43 other tissues and cell lines (Fig. 5a). The FPKM for *lncRNA-001762* in HSC myofibroblasts is 2.9 and increases to 5.4 with TGF-β treatment, whereas the mean in all other samples is 0.13 with maximum expression of 0.85 in human umbilical vein endothelial cells (HUVECs). The restriction in expression to HSCs is further illustrated by visualizing the RNA-seq reads mapped to *lncRNA-001762* in the HUVEC cell line and testis (Fig. 5b). These examples were chosen because they are the cell line and primary tissue with the next highest expression of *lncRNA-001762* compared with HSC myofibroblasts. Whole liver tissue and the HepG2 cells express even lower levels of *lncRNA-001762*, which suggests that this lncRNA is likely restricted in expression to HSCs in the liver and is not induced in hepatocellular carcinoma.

### lncRNAs expressed in HSCs are enriched in ECM networks

Co-expression network analyses have been used widely to predict the functions of unknown coding and noncoding genes [93–100] based on the expectation that genes with similar expression patterns across multiple tissues, cell types, or conditions share similar functions or are involved in related biological processes [101, 102]. Therefore, we conducted co-expression network analyses across the 37 primary human tissues, six ENCODE cell lines, and the HSCs described in this study using expression profiles for all annotated genes expressed in at least one tissue or cell type and the lncRNAs identified in this study.

**Fig. 6:**
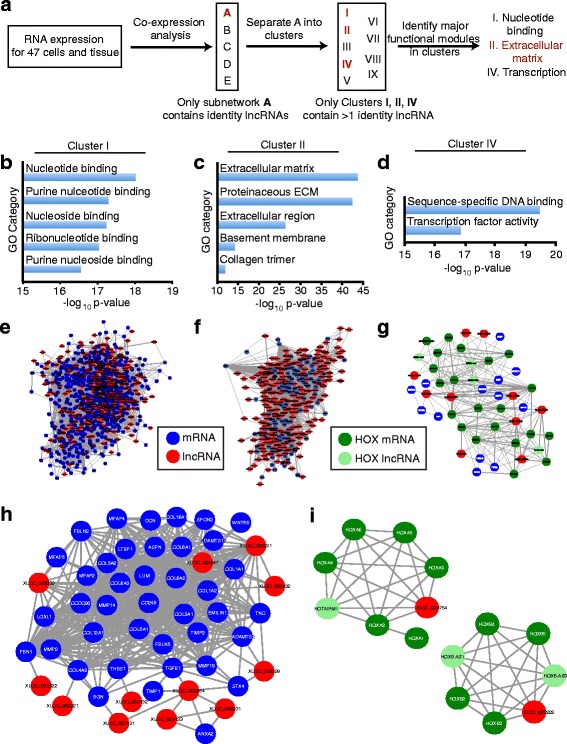
lncRNAs discovered in HSCs form networks with ECM proteins. **a** Schematic of co-expression analysis. Expression of all protein-coding genes as well as the lncRNAs expressed in HSCs were analyzed in HSCs, 37 primary tissues, and six ENCODE cell lines. Co-expression analysis (Spearman coefficient >0.7) identified five main subnetworks. Only subnetwork A contained HSC identity lncRNAs. Subnetwork A was divided into nine main clusters. Only three clusters (*red*) contained more than one HSC identity lncRNA. Gene ontology (GO) analysis was performed to identify the most enriched categories for each of these three clusters. The extracellular matrix was significantly enriched in cluster II. The top GO categories identified for cluster I (**b**), cluster II (**c**), and cluster IV (**d**) are shown. The -log10 *p* values are displayed on the x-axis. **e** The protein-coding genes from cluster I that are contained in the GO category of nucleotide binding are displayed along with all HSC lncRNAs contained in cluster I that are connected to the nucleotide-binding protein-coding genes by one edge (*gray line*s). mRNAs (*blue*) and lncRNAs (*red*) are represented as *circles*. **f** The protein-coding genes from cluster II that are contained in the GO category of ECM are displayed along with all HSC lncRNAs contained in cluster II that are connected to the ECM proteins by one edge. **g** The entire network for cluster IV is displayed, including all protein-coding genes and HSC lncRNA genes. This cluster contains multiple HOX genes (*dark green circles*) and lncRNAs associated with HOX genes (*HOX lncRNA*, *light green circles*). **h** The HSC identity lncRNAs and the ECM proteins in cluster II that are connected by one edge are shown. HSC identity lncRNAs were defined as occupied by SMAD3, associated with super-enhancers, and restricted in expression to HSCs. **i** Two super-enhancers regulate genes encoding mRNAs and lncRNAs in cluster IV. Genes encoding mRNAs and lncRNAs located within a super-enhancer and connected by one edge are shown for two super-enhancers

We identified 169 subnetworks containing at least two nodes where each node represents a protein-coding gene or an lncRNA and the edge between two nodes indicates that the two genes are co-expressed with a Spearman-correlation coefficient >0.7 (*p* < 4e-7; Fig. 6a). Most of the identified subnetworks were small and only five contained more than five nodes. To identify the subnetworks that would provide the most information about lncRNAs acting in HSCs, each subnetwork was scanned for HSC identity lncRNAs that were defined as lncRNAs proximal to super-enhancers, occupied by SMAD3, and restricted in expression to HSCs (Additional file 10: Table S9). Thirty-three lncRNAs met these criteria and 21 were contained in subnetwork A. HSC identity lncRNAs were not found in any of the other subnetworks. Subnetwork A contains 18,002 nodes and was decomposed to identify smaller clusters that may provide further insight into the function of lncRNA genes. Nine clusters were identified based on their connection structure and correlation value (see “*Methods*” for details) and three clusters contained more than one HSC identity lncRNA. Cluster I contained 3950 nodes, including two identity lncRNAs and 332 lncRNAs expressed in HSCs. Cluster II contained 1865 nodes, including nine identity lncRNAs and 314 lncRNAs expressed in HSCs. Cluster IV contained 48 nodes, including two identity lncRNAs and seven lncRNAs in HSCs.

We then performed gene ontogeny (GO) enrichment analysis on clusters I, II, and IV to identify cellular processes or functions that were key components of each cluster. Cluster I was heavily enriched in nucleotide binding (Fig. 6b), cluster II in ECM (Fig. 6c) and cluster IV in DNA binding factors (Fig. 6d). We visualized cluster I to show the size of the network and the relative composition of mRNA and lncRNA genes by mapping all protein-coding genes from cluster I that were contained in the nucleotide-binding category and all the lncRNAs in cluster I within one edge of these protein-coding genes. This nucleotide-binding functional module is composed of 521 genes encoding mRNA (blue) and 262 genes encoding lncRNAs (red) (Fig. 6e). We visualized the ECM functional module in cluster II by mapping all protein-coding genes from cluster II that were contained in the ECM category and all the lncRNAs in cluster II within one edge of these protein-coding genes. This ECM module is more enriched for lncRNAs, being composed of 110 genes encoding mRNAs and 211 genes encoding lncRNAs (Fig. 6f). We visualized cluster IV by showing all protein-coding genes and lncRNA genes in that cluster. Cluster IV is enriched in HOX transcription factor genes (dark green) and known lncRNA genes associated with HOX loci (light green) (Fig. 6g). Lists of individual genes encoding mRNAs and lncRNAs and the co-expression pairs related to each GO category (nucleotide binding in cluster I, ECM in cluster II and Cluster IV) are included in Additional file 11: Table S10.

The most significant finding from this analysis is that a module of genes encoding mRNAs and lncRNAs is highly associated with the ECM, whose production by HSC myofibroblasts is the primary cause of liver fibrosis and progression to liver failure in chronic liver disease [9, 10]. To exclude the possibility that the presence of protein-coding genes and lncRNAs highly enriched in HSCs skewed the co-expression network analysis, we repeated the analysis without the HSC data. This analysis also yielded the ECM module within cluster II, demonstrating that this is a robust functional module even in the absence of HSC expression data (Additional file 3: Figure S6).

Twelve HSC identity lncRNAs were present within one edge of protein-coding genes in cluster II that were associated with ECM (Fig. 6h). This sub-cluster is highly enriched in genes related to liver fibrosis, including 11 collagen genes and genes encoding *TGFB1*, matrix metalloproteinases, tissue inhibitors of metalloproteinases, and lysyl oxidase like proteins. These findings suggest that the 12 identity lncRNAs in this sub-cluster likely contribute to ECM production and liver fibrosis.

We then asked if coordinated expression of coding and noncoding genes within super-enhancers could explain the structure of the co-expression networks. Cluster I and cluster II contained 30 paired genes encoding lncRNAs and mRNAs that were co-expressed and located in the same super-enhancers, but these examples were almost entirely associated with divergently transcribed genes that were located in a super-enhancer. In contrast, cluster IV did contain two super-enhancers where multiple mRNAs and lncRNAs were co-expressed (Fig. 6i). While cluster IV does contain examples of co-expressed genes within super-enhancers, association within super-enhancers appears to account for only a small fraction of co-expressed genes.

The majority of lncRNAs expressed in HSC myofibroblasts are divergently transcribed from genes that encode proteins (Fig. 2a). We found that over 90 % of divergent lncRNAs expressed in HSCs were paired with protein-coding genes that are also expressed in HSCs (Additional file 12: Table S11). We analyzed expression of each lncRNA and the paired protein-coding gene across 37 primary tissues [75] and the six tier 1 and tier 2 cell lines from the ENCODE project [92] and found that only 14 % of these pairs are co-expressed across tissues (Spearman correlation >0.7, *p* < 4e-7; Additional file 13: Table S12). Thus, in many cell types where a protein-coding gene is expressed, the divergent lncRNA identified in HSCs is silent.

### lncRNAs expressed in fetal HSCs exhibit expression patterns similar to those in adult HSCs

**Fig. 7:**
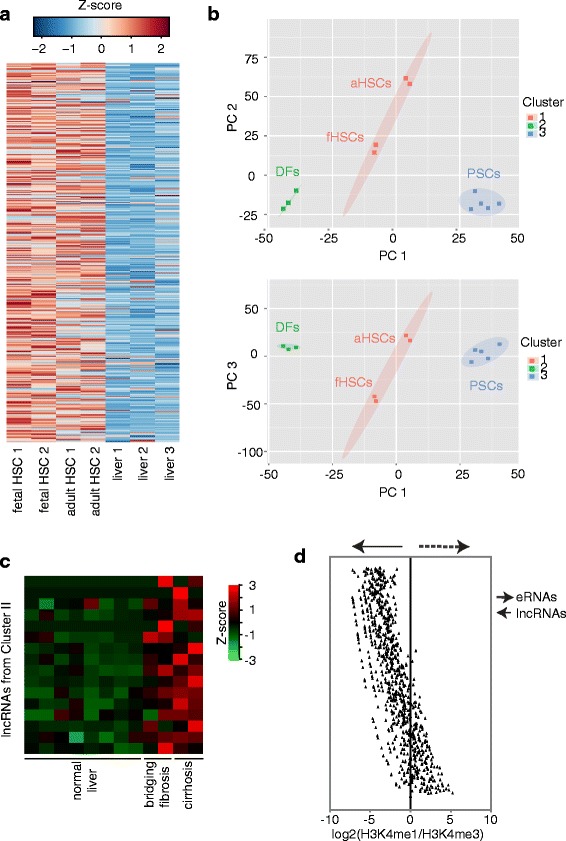
lncRNA expression across myofibroblasts from different tissues. **a** Expression of HSC-specific lncRNAs (Fig. 4) was analyzed in fetal HSCs, adult HSCs, and liver. **b** PCA and k-mean clustering show that adult and fetal HSC myofibroblasts cluster together (*aHSCs* and *fHSCs*, *red*) compared with pancreatic stellate cell myofibroblasts (*PSCs*, *blue*) and dermal fibroblasts (*DFs*, *green*) based on lncRNA expressions. Principal component (PC)2 versus PC1 is displayed on the *top* and PC3 versus PC1 is displayed on the *bottom*. **c** Expression of lncRNAs contained in the ECM network (Fig. 6f) was analyzed across eight normal liver samples, two with bridging fibrosis and two with cirrhosis [75]. Sixteen lncRNAs from cluster II (*y-axis*) were found to be enriched in fibrosis and cirrhosis compared with normal livers. Each *row* represents expression of one lncRNA and the liver histology is indicated on the *x-axis*. The Z-score is shown in the upper right. **d** Identification of lncRNAs that meet criteria of unidirectional eRNAs. The ratio of H3K4me1/H3keme3 was calculated at the TSS (±0.5 kb) of the selected lncRNAs. A ratio >1.2 was used to identify eRNAs (indicated by *dotted arrow*) [80]

The lncRNAs analyzed in this study were defined in human fetal HSCs myofibroblasts and we next asked if these findings also apply to adult HSC myofibroblasts. We performed RNA-sequencing to quantify expression of lncRNAs in primary human HSCs that were transdifferentiated into HSC myofibroblasts by ex vivo culture. Over 96 % (3566 out of 3691) of lncRNAs detected in fetal HSC myofibroblasts were also detected in adult HSC myofibroblasts (Additional file 14: Table S13). The 435 lncRNAs that were uniquely enriched in fetal HSC myofibroblasts (Fig. 4) were also highly enriched in adult HSC myofibroblasts (Fig. 7a). We then asked if these lncRNAs were specific to HSC myofibroblasts or were also expressed in myofibroblasts originating in other tissues. We found that pancreatic stellate cells [103] and dermal fibroblasts [75] also expressed many lncRNAs in common with fetal HSC myofibroblasts (Additional file 3: Figure S7a, Additional file 14: Table S13). However, lncRNA expression in fetal HSC myofibroblasts was more closely associated with lncRNA expression in adult HSC myofibroblasts compared with pancreatic stellate cell myofibroblasts (*p* < 1.23 e-7) or dermal fibroblasts (*p* < 8.8e-6) and is also shown by PCA (Fig. 7b). This association was also present when taking into account expression of both lncRNAs and protein-coding genes (Additional file 3: Figure S7b). In addition, adult HSC myofibroblasts stimulated with TGF-β signaling showed similar patterns of gene induction and repression compared with fetal HSC myofibroblasts treated with TGF-β (Additional file 3: Figure S7c, Additional file 15: Table S14).

Cluster II (Fig. 6f) contains the lncRNAs that form a network with ECM proteins. Adjusting this network to contain lncRNAs expressed in adult HSC myofibroblasts led to removal of only four lncRNAs (Additional file 3: Figure S7d). We then analyzed expression of the lncRNAs in the ECM module across normal human liver tissue, bridging fibrosis, and cirrhosis. This analysis identified 16 lncRNAs that were expressed in both fetal and adult HSC myofibroblasts and were significantly enriched in human liver fibrosis and cirrhosis compared with normal livers (Fig. 7c), suggesting that induction of these lncRNAs is associated with progression of human liver fibrosis. It also indicates that the ECM module is enriched in lncRNAs induced in bridging fibrosis and cirrhosis (7.6 %) compared with the total lncRNA pool in HSCs (1.4 %).

### A small fraction of the noncoding RNAs identified in HSCs can be classified as unidirectional eRNAs

lncRNAs were identified in this study by the presence of polyadenylation and size greater than 200 nt. eRNAs are noncoding RNAs (ncRNAs) transcribed from enhancers and tend not to be polyadenylated or spliced [104]. They can be transcribed unidirectionally or bidirectionally and unidirectional eRNAs can be polyadenylated [105]. The functional differences between polyadenylated unidirectional eRNAs and lncRNAs transcribed from enhancers are not clearly understood [106], but the abundance of the chromatin mark H3K4me1 compared with H3K4me3 has been used to classify these ncRNA transcripts [22, 80]. To determine the fraction of lncRNAs identified in this study that overlap with polyadenylated unidirectional eRNAs, we analyzed ChIP-seq data of H3K4me1 and H3K4me3 in immortalized human induced fibroblasts (hiF-Ts) [107]. Of the 1042 lncRNAs identified in HSCs and classified as enhancer-associated or intergenic (Fig. 2a), 851 were also expressed in hiF-Ts. Using the H3K4me1:H3K4me3 ratio >1.2 as the threshold, we found that 181 of 851 ncRNAs (21 %) meet the definition of eRNAs (Fig. 7d; Additional file 16: Table S15) [80] and 90 of these loci encode only single exon ncRNAs. It is not clear if these unidirectional eRNAs may have different activities to lncRNAs, but eRNAs accounted for only 5.6 % of lncRNAs in the ECM functional module (Fig. 6f) and removal of these eRNAs led to loss of two additional protein-coding genes from the ECM module (Additional file 3: Figure S7d and Additional file 11: Table S10).

## Discussion

HSCs are the primary cell type responsible for liver fibrosis and liver failure in chronic liver disease. While many protein-coding genes that regulate HSC function have been described, the diversity of lncRNA expression in HSCs and the biological pathways they affect are unknown. This study was performed to define the lncRNAs expressed in human HSCs and to predict those that are likely to regulate the fibrotic process. Identification of lncRNAs uniquely enriched in HSCs will provide potential targets to inhibit the progression of fibrosis without affecting other cell types in the liver.

We also find that many lncRNAs are expressed divergently from protein-coding genes, as previously described [55, 58]. While our analysis does not address co-expression of the paired protein-coding and lncRNA genes at the single cell level [108], it does suggest that the lncRNAs and their divergent protein-coding genes are usually both expressed is HSCs. In contrast, a minority of these paired lncRNA and protein-coding genes are co-expressed across different cell types. Thus, while a protein-coding gene may be expressed in many different cell types, the expression of its paired, divergent lncRNA appears to be more restricted, suggesting that there are cell type-specific levels of lncRNA gene regulation independent of the transcriptional control of the divergent protein-coding gene.

Our analysis also identified 195 lncRNAs that are directly affected by TGF-β signaling as indicated by SMAD3 occupancy [90] and change in expression in response to TGF-β signaling. In addition, lncRNAs directly regulated by TGF-β signaling are enriched in super-enhancers, which suggests that the lncRNAs controlled by TGF-β signaling may play key roles in HSC function and fibrosis. TGF-β is a key activator of fibrosis [2] and this analysis was performed on HSC myofibroblasts that underwent transdifferentiation ex vivo as a model to identify human lncRNAs regulated by TGF-β signaling. Now that these lncRNAs have been defined, it will be important to identify and characterize those that are induced in human liver disease.

Genes that control cell identity tend to be lineage-restricted [109–114]. To identify additional lncRNAs that may be key contributors to fibrosis progression, we defined the lncRNAs enriched in HSC myofibroblasts. We identified over 400 lncRNAs that are restricted in expression to HSCs compared with 43 other tissues and cell types. This analysis provides a more accurate representation of cell-type specificity than the identification of HSC lncRNAs that were not previously annotated because ab initio assembly of lncRNA transcripts has not been performed in all the tissues and cell types we were able to analyze. By defining the lncRNAs that were HSC-specific, bound by SMAD3, and associated with super-enhancers, we were able to focus on a small set of lncRNAs that are most likely to control HSC myofibroblast cell identity, which we refer to as identity lncRNAs. Tracing these lncRNAs through the co-expression network analysis allowed us to focus on the networks containing lncRNAs most relevant to HSC function.

Co-expression network analysis revealed that lncRNAs are co-expressed with protein-coding genes that regulate production of ECM proteins. Over half the genes in this network are lncRNAs and the network also includes numerous collagen genes, whose products make up the fibrotic scar, as well as proteins that are responsible for crosslinking and remodeling the ECM. Co-expression analysis was repeated in the absence of HSC expression data and confirmed that the ECM network is present. These findings indicate that the ECM network represents a robust co-expression network that is not identified solely based on high expression of genes in HSCs. In addition, a subset of the lncRNAs in the ECM network is induced in human liver fibrosis (Fig. 7c), suggesting that they are associated with human liver disease. HSCs make up only 5–10 % of the cells in the liver [115] and additional lncRNAs are likely to be induced in HSCs during the progression of fibrosis that were not detected in whole liver samples due to lower levels of expression.

We found that lncRNA expression was highly conserved between fetal and adult HSC myofibroblasts. In addition, many lncRNAs were also shared between HSC myofibroblasts, pancreatic stellate cell myofibroblasts, and dermal fibroblasts. Analysis of lncRNAs alone or lncRNAs and protein-coding genes together revealed that fetal HSC myofibroblasts were more closely related to adult HSC myofibroblasts than to the other cell types analyzed. This study focused on the identification of lncRNAs in HSC myofibroblasts but also suggests that understanding the role of lncRNAs in HSC myofibroblasts may lead to insight into the function of lncRNAs in myofibroblasts from other tissues.

## Conclusions

We provide the first comprehensive catalog of lncRNAs expressed in human HSCs and demonstrate that the lncRNAs identified are relevant to human liver disease. We discovered more than 3600 lncRNAs, including approximately 40 % that have not been described in other cell types and greater than 400 that are uniquely enriched in HSCs compared with 43 other tissues and cell types. We analyzed the genomic location, chromatin modifications, response to differentiation and signaling, and expression across different tissues and cell types to identify lncRNAs that are likely to be involved in HSC function and fibrosis. This analysis provides a resource for future studies to investigate lncRNA function in liver disease and fibrosis.

## Additional files

Additional file 1: Table S1. GTEx samples used in expression analyses. (XLS 40 kb) Additional file 2: Table S2. GTEx ID and pathology reads of eight normal livers, two cirrhosis livers, and two bridging-fibrosis livers. (XLS 22 kb) Additional file 3: Supplemental Figures and legends. (PDF 3744 kb) Additional file 4: Table S3. lncRNA transcripts and loci found in HSC myofibroblasts. (XLS 1520 kb) Additional file 5: Table S4. The genomic locations of super-enhancers. (XLS 60 kb) Additional file 6: Table S5. lncRNA transcripts and loci identified in HSC myofibroblasts, HSC myofibroblasts during serum starvation, and HSC myofibroblasts after TGF-β stimulation. (XLS 3456 kb) Additional file 7: Table S6. Expression levels of all lncRNA loci in each condition of HSC. (XLS 408 kb) Additional file 8: Table S7. lncRNAs directly induced/repressed by TGF-β signaling pathway. (XLSX 53 kb) Additional file 9: Table S8. Expression profiles of HSC-specific lncRNAs across HSC myofibroblasts, TGF-β HSCs, and 43 human tissues or cell types. (XLS 389 kb) Additional file 10: Table S9. lncRNAs’ association with SMAD3, super-enhancers, and restricted expression in HSCs. (XLS 312 kb) Additional file 11: Table S10. Co-expression pairs and gene lists of major functional modules in clusters I, II and IV. (XLS 988 kb) Additional file 12: Table S11. Expression levels of lncRNAs and their divergent paired coding genes in HSC myofibroblasts. (XLS 310 kb) Additional file 13: Table S12. Expression profile of divergently paired protein-coding genes and lncRNAs that are co-expressed across 43 human tissues and cell types. (XLSX 533 kb) Additional file 14: Table S13. The mean expression levels in FPKM of lncRNAs in adult HSCs, PSCs, dermal fibroblasts, and livers. (XLSX 328 kb) Additional file 15: Table S14. The expression levels in FPKM of lncRNAs in TGF-β starvation and TGF-β treatment adult HSCs. (XLSX 250 kb) Additional file 16: Table S15. H3K4me1:H3K4me3 ratio of the 851 lncRNAs in hiF cells. (XLS 78 kb)

## Abbreviations

ACTA2: actin, alpha2 smooth muscle
ChIP: chromatin immunoprecipitation
DMEM: Dulbecco’s modified Eagle medium
ECM: extracellular matrix
ENCODE: Encyclopedia of DNA Elements
eRNA: enhancer RNA
FCS: fetal calf serum
FPKM: fragments per kilobase of transcript per million fragments mapped
GEO: Gene Expression Omnibus
GO: Gene Ontology
GTEx: Genotype-Tissue Expression
HCC: hepatocellular carcinoma
hiF-T: human induced fibroblast
HSC: hepatic stellate cell
HUVEC: human umbilical vein endothelial cell
kb: kilobase
lncRNA: long noncoding RNA
logLR: log-likelihood ratio
LOX: lysyl oxidase
LOXL2: lysyl oxidase like 2
MCL: Markov Clustering
mRNA: messenger RNA
ncRNA: noncoding RNA
nt: nucleotide
P/S: penicillin/streptomycin
PC: principal component
PCA: principal component analysis
snoRNA: small nucleolar RNA
TGF-β: transforming growth factor beta
TSS: transcription start site
UCSC: University of California, Santa Cruz
