# Supplementary material for: Long noncoding RNAs expressed in human hepatic stellate cells form networks with extracellular matrix proteins
Source: Genome Med. 2016 Mar 23;8:31. doi: 10.1186/s13073-016-0285-0 (PMC4804564; doi:10.1186/s13073-016-0285-0)

Supplemental Figure legends

Supplemental Figure S1, related to Figure 1: Protein-coding potential calculations. All lncRNA transcripts from HSC myofibroblasts (Fig. 1D) were analyzed for protein-coding potential using HMMER (purple) and CPAT (green). The lncRNAs determined to have low protein-coding potential by both methods were considered to have low coding potential. The number of transcripts in each category is indicated.

Supplemental Figure S2, related to Figure 2: Length of lncRNA and protein-coding genes. **(A)** The gene lengths of lncRNAs and protein-coding genes expressed in HSC myofibroblasts are shown (x-axis). The longest isoform in each locus was chosen for analysis. LncRNA genes are shown in red and protein-coding genes are shown in blue. The dotted lines indicate median gene length for lncRNAs (red) and protein-coding genes (blue). **(B)** The transcript lengths of lncRNAs genes and mRNAs are shown (x-axis). The longest isoform for each lncRNA and mRNA were used for analysis.

Supplemental Figure S3, related to Figure 3: Change in gene expression and conservation of lncRNAs. **(A)** Genes regulated by TGF-β signaling are induced with TGF-β treatment of HSC myofibroblasts. Protein-coding gene expression was quantified in low TGF-β conditions and after treatment of HSCs with TGF-β for 16 hours (Fig. 3A). The log2 fold change in expression is shown for each protein-coding gene in two replicates treated with TGF-β relative to the mean expression level of the gene in two replicates in low TGF-β conditions. The list on the right indicates selected genes that are induced by treatment with TGF-β. **(B)** The number of lncRNA loci assembled in HSC myofibroblasts, HSC myofibroblasts following serum starvation (low TGF-β) and HSC myofibroblasts treated with TGF-β following serum starvation are shown. All the lncRNA loci identified in any condition were then combined to generate the list of 3692 lncRNA expressed in HSCs. **(C)** The fraction of total lncRNA loci identified in this

study that were previously annotated is shown. Three different lncRNA databases (NONCODE (22), fRNAdb (23) and Human Body Map Linc (24) shown in x-axis) were analyzed. An lncRNA was considered previously annotated if the lncRNA overlapped by one or more nucleotides on the same strand with an annotated lncRNA. The fraction of HSC lncRNAs that are annotated in any database (combined databases) is shown on the far right.

Supplemental Figure S6, related to Figure 6: Constructing co-expression networks without protein-coding genes and lncRNAs expressed in HSCs. Subnetwork A was assembled as in Fig 6A except that the expression data from HSC mRNAs and lncRNAs was not used. Cluster II was still identified without the HSC input and was enriched for ECM. The ECM proteins from Cluster II are displayed as well as all the HSC lncRNAs that are within one edge of these protein-coding genes.

Supplemental Figure S7, related to Figure 7: Comparison of fetal HSCs with adult HSCs and other myofibroblasts. (A) Expression profile of the 435 HSC-specific lncRNAs across fetal HSC myofibroblasts (fHSCs), adult HSC myofibroblasts (aHSCs), pancreatic stellate cell myofibroblasts (PSCs), dermal fibroblasts (DFs) and livers. **(B)** Principle Component Analysis and K-mean clustering classified fetal and adult HSCs into one cluster (red), PSCs into a second cluster (blue), and DFs into a third cluster (green) according to the expression levels of protein-coding genes and lncRNAs. Principle component (PC) 1 vs PC 2 are displayed on the left and PC1 vs PC3 are displayed on the right. **(C)** TGF-β signaling has a similar affect on lncRNA expression in adult and fetal HSCs. We identified 139 lncRNAs in fetal HSC myofibroblasts that were induced and 242 lncRNAs that were repressed by TGF-β signaling (Figure 3B). The change in expression in adult HSC myofibroblasts under identical treatment

conditions is shown for the same lncRNAs. (**D)** The ECM module (Figure 6F) is shown after excluding eRNAs. Protein-coding genes are shown in blue and lncRNAs in red.

Supplemental Figure S1

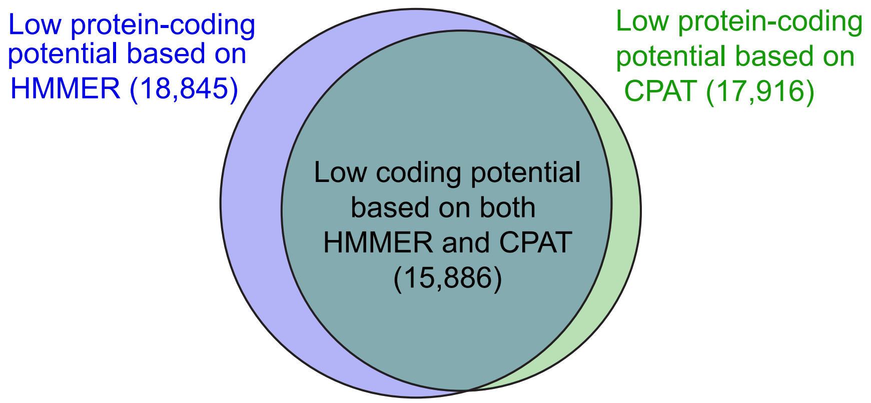

Supplemental Figure S2

A

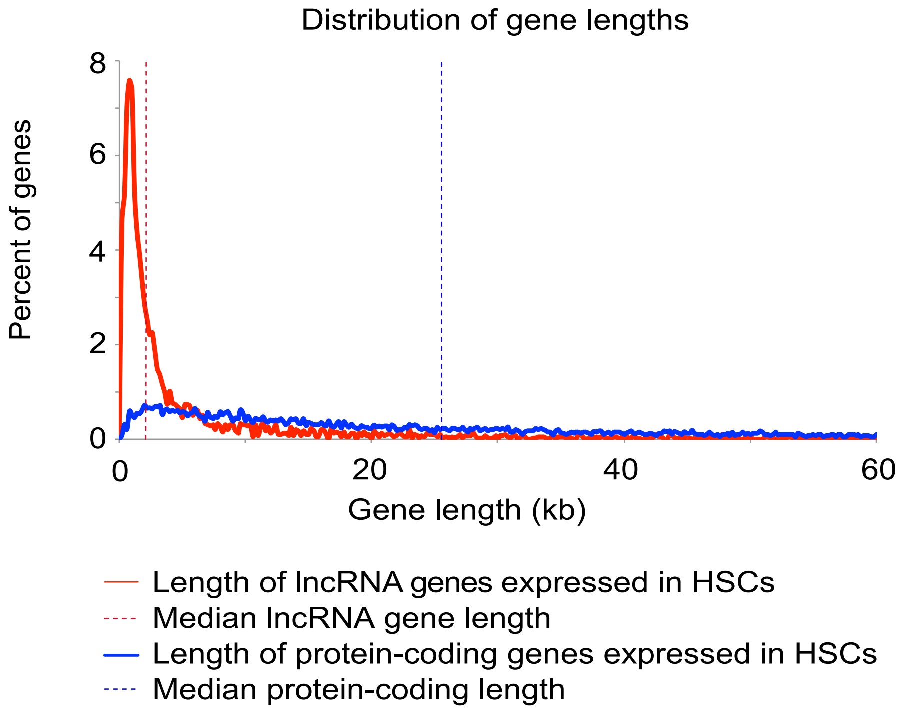

B

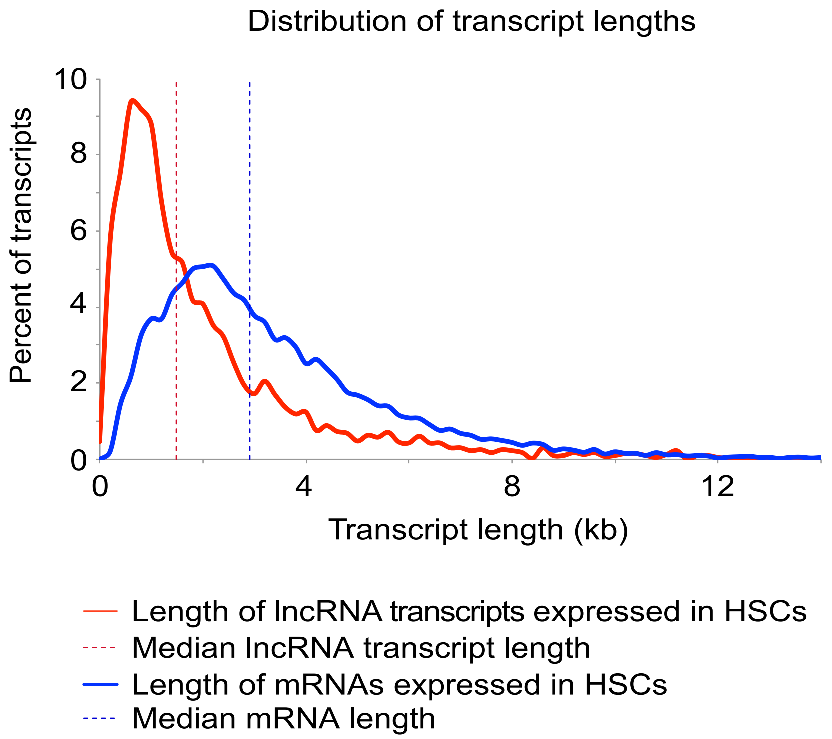

Supplemental Figure S3

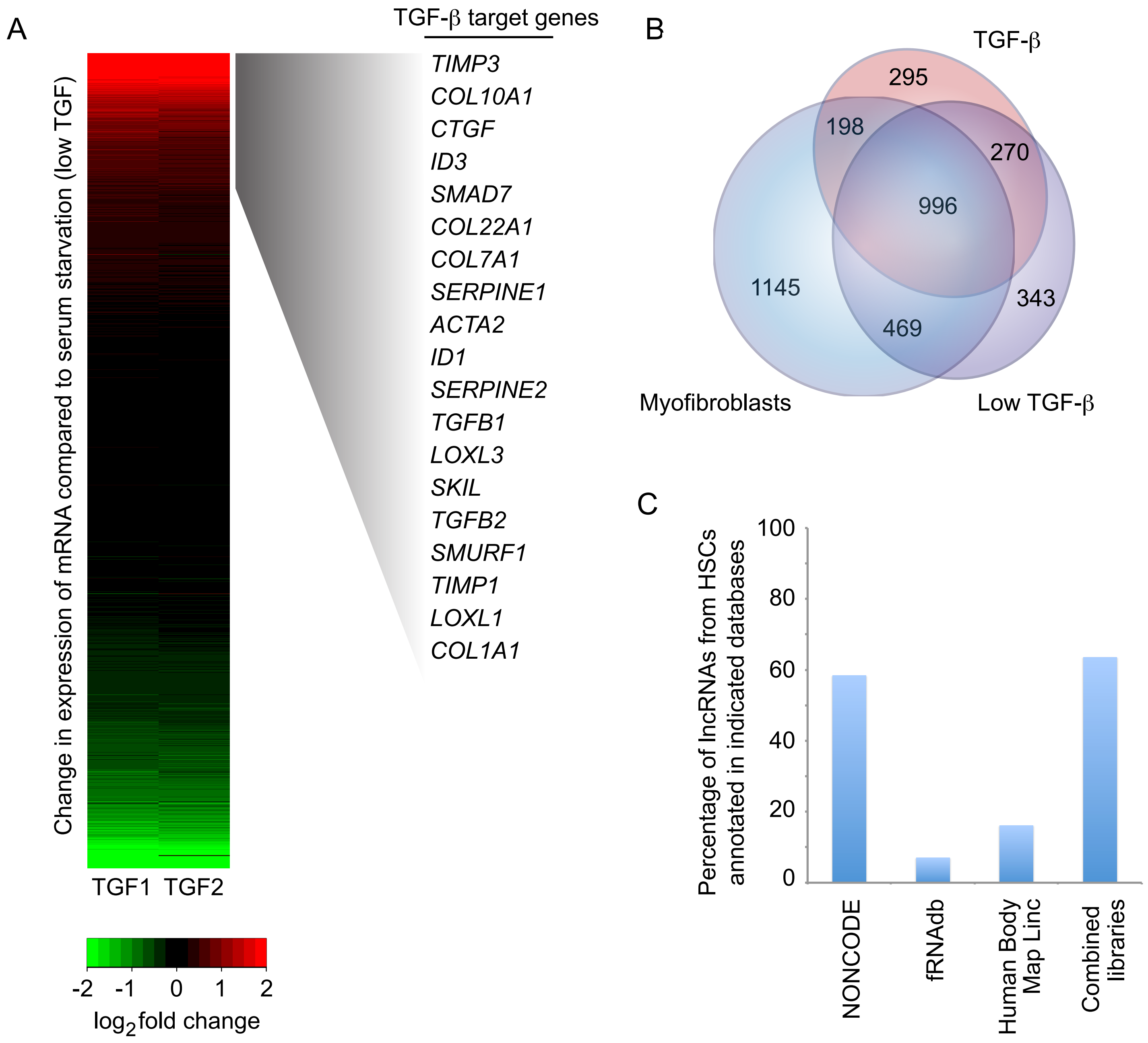

Supplemental Figure S6

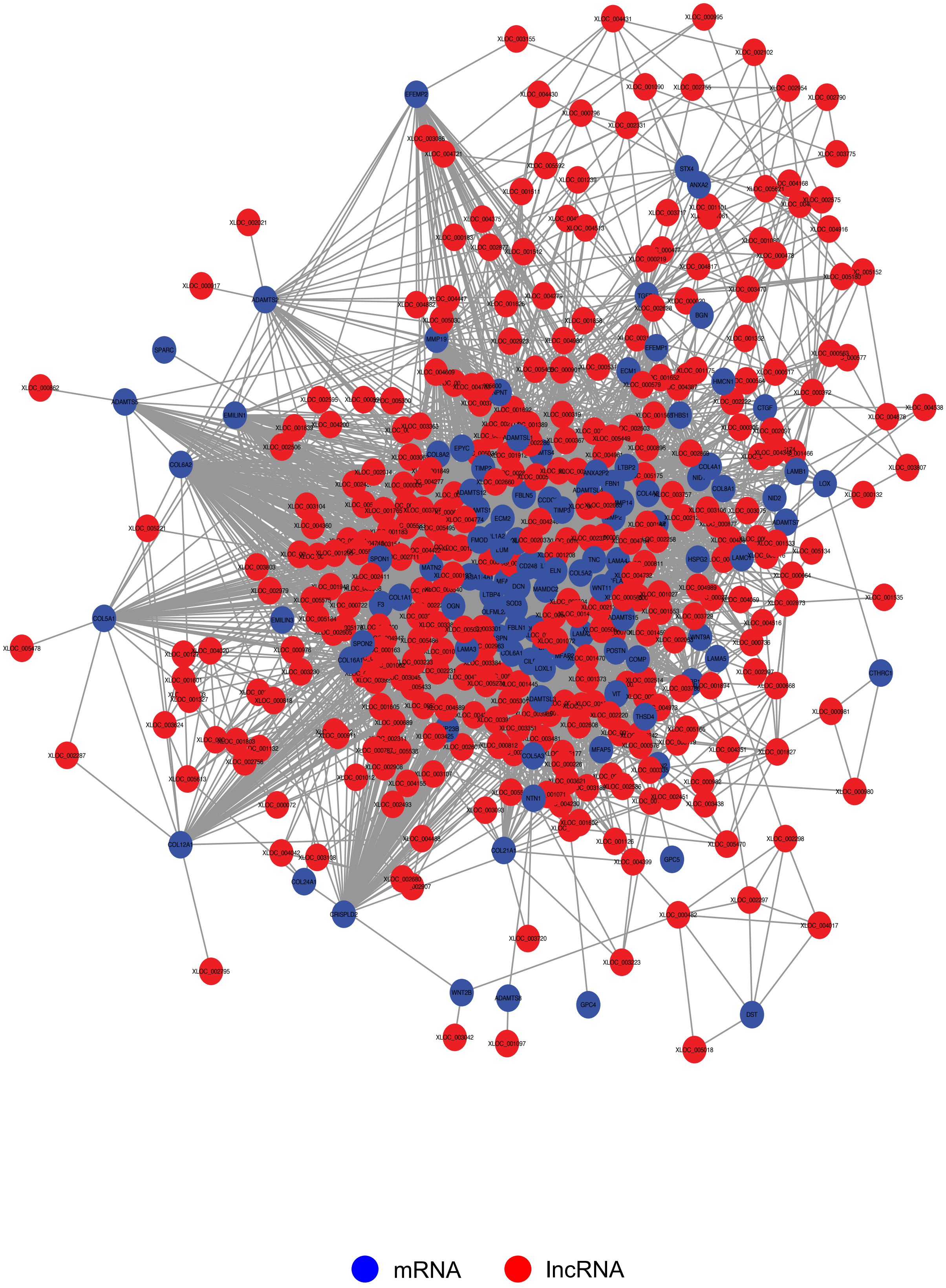

Supplemental Figure S7

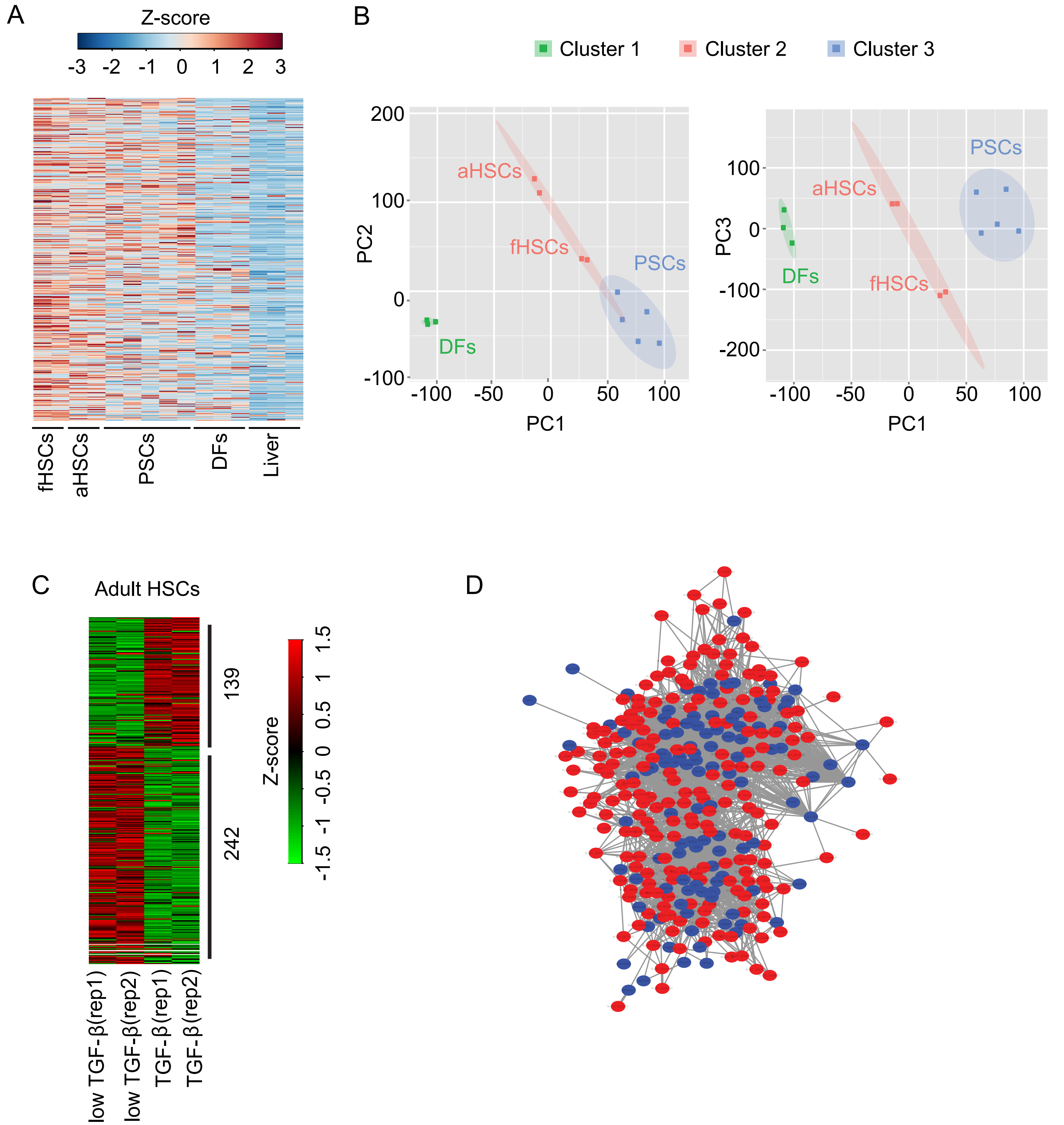

Supplement: Additional file 3: — Supplemental Figures and legends. (PDF 3744 kb) [file 13073_2016_285_MOESM3_ESM.pdf]
